# Emergency knowledge in one rescue team of power network

**DOI:** 10.1186/1471-227X-12-S1-A4

**Published:** 2012-12-18

**Authors:** MENG Fanshan, ZHAO Yulan, DAI Dongme

**Affiliations:** 1 Emergency Department, the 88th Hospital of PLA, Taian 271000, Shandong, China

## Objective

To investigate the mastery of emergency knowledge in one rescue team of power network in Taian.

## Methods

We investigated emergency knowledge in one rescue team of power network in Taian by questionnaires, which include CPR skill, hemostasis, treatment of respiratory tract obstruction, drowning, electric shock, intoxication and spinal fractures.

## Results

The members of the team have a little general emergency knowledge, especially in CPR and treatment of spinal rupture, but they could not treat them properly and needed further training, and most of them would like to gain the emergency knowledge and perform it in case of emergency.

## Conclusion

The rescue team of power network knew a little emergency knowledge, but they needed further training. They could be a desirable group for emergency training.

